# Molecular Mechanisms of Hypertensive Nephropathy: Renoprotective Effect of Losartan through Hsp70

**DOI:** 10.3390/cells10113146

**Published:** 2021-11-12

**Authors:** Valeria Victoria Costantino, Andrea Fernanda Gil Lorenzo, Victoria Bocanegra, Patricia G. Vallés

**Affiliations:** 1 IMBECU-CONICET, Instituto de Medicina y Biología Experimental de Cuyo—Consejo Nacional de Investigaciones Científicas y Técnicas, Mendoza 5500, Argentina; valecostantino@hotmail.com (V.V.C.); mvbocanegra@gmail.com (V.B.); 2 Área de Biología Celular, Departamento de Morfofisiología, Facultad de Ciencias Médicas, Universidad Nacional de Cuyo, Mendoza 5500, Argentina; 3 Área de Fisiopatología, Departamento de Patología, Facultad de Ciencias Médicas, Universidad Nacional de Cuyo, Mendoza 5500, Argentina; andreafernandagillorenzo@gmail.com

**Keywords:** hypertension, nephrosclerosis, epithelial–mesenchymal transition, Hsp70 chaperone, proximal tubule epithelial cells

## Abstract

Hypertensive nephrosclerosis is the second most common cause of end-stage renal disease after diabetes. For years, hypertensive kidney disease has been focused on the afferent arterioles and glomeruli damage and the involvement of the renin angiotensin system (RAS). Nonetheless, in recent years, novel evidence has demonstrated that persistent high blood pressure injures tubular cells, leading to epithelial–mesenchymal transition (EMT) and tubulointerstitial fibrosis. Injury primarily determined at the glomerular level by hypertension causes changes in post-glomerular peritubular capillaries that in turn induce endothelial damage and hypoxia. Microvasculature dysfunction, by inducing hypoxic environment, triggers inflammation, EMT with epithelial cells dedifferentiation and fibrosis. Hypertensive kidney disease also includes podocyte effacement and loss, leading to disruption of the filtration barrier. This review highlights the molecular mechanisms and histologic aspects involved in the pathophysiology of hypertensive kidney disease incorporating knowledge about EMT and tubulointerstitial fibrosis. The role of the Hsp70 chaperone on the angiotensin II–induced EMT after angiotensin II type 1 receptor (AT_1_R) blockage, as a possible molecular target for therapeutic strategy against hypertensive renal damage is discussed.

## 1. Introduction

Hypertension affects approximately 30% of the general population. Hypertensive kidney disease is considered one of the consequences of long-term and poorly controlled hypertension. Hypertensive kidney disease is the second leading cause of end-stage renal disease (ESRD) after diabetes [1]. Most hypertensive patients develop mild-to-moderate hypertensive nephrosclerosis. Nevertheless, the percentage of patients that develop ESRD dramatically increases when blood pressure (BP) values are uncontrolled for a long time, or kidney disease preexists. In most cases of concomitant hypertension and chronic kidney disease (CKD), the sequence of events (i.e., which came first, CKD or hypertension) cannot be established. For a long time attention has been given to the capillary tuft damage causing nephrosclerosis and hyalinosis, and to the activity of the renin–angiotensin system (RAS) [1,2]. However, recently, molecular mechanisms and other histologic aspects have been included in the pathophysiology of hypertensive kidney disease including the injury of tubular cells that induces epithelial–mesenchymal transition (EMT) and tubulointerstitial fibrosis. Several intracellular signaling systems are involved in EMT and renal fibrosis. In the kidney, angiotensin II actively participates in renal fibrosis, in part mediated by TGFβ (transforming growth factor β) [3]. Previous studies have demonstrated that the MAPK pathway is required for TGFβ-mediated EMT and cell migration [4]. In addition, activation of small Rho GTPases is a key step in EMT [5]. It is now clear that angiotensin II signals through the AT_1_-ERK (extracellular signal-regulated)/p38 MAP (mitogen-activated protein) kinases, TGF-β/Smad2/3, and NF-κB (nuclear factor κ-light-chain-enhancer of activated B cells) pathways to promote renal oxidative stress and mediate epithelial mesenchymal transdifferentiation [6,7]. 

A cell protective mechanism against oxidative stress includes the production of highly conserved proteins called heat shock proteins (Hsps). Hsps are intracellular proteins that respond to cellular stress and act as chaperones of other ‘client’ peptides to which they bind to prevent their irreversible aggregation and promote their correct folding, physiological assembly and intracellular trafficking. The Hsps interact in a complex network of ATP-independent and ATP-dependent chaperones that are primarily cytoprotective and are critically involved in the functional preservation of many regulatory pathways. Once their function is accomplished, the Hsps dissociate from their client proteins [8]. Hsps are classified by their molecular weight and grouped in families. The various forms of HSP70 family consist of an N-terminal 43-kDa nucleotide-binding domain (NBD) and a 27-kDa C-terminal, which serves as a substrate-binding domain (SBD) with both parts being connected by a highly conserved linker. ATP binds to the NBD, while misfolded client proteins interact with the SBD. This chaperone is assisted by co-chaperones. Accordingly, there are at least two conceptual ways of targeting HSP70 family members: block ATPase activity or change protein-protein interactions with co-chaperones [9].

This review focuses on emerging knowledge of biochemical and molecular mechanisms underlying the kidney damage in primary (essential) hypertension. We also provide data on the role of Hsp70 as a modulator of losartan effect on the cytoskeleton, intercellular junctions stabilization and the epithelial–mesenchymal transition in proximal tubular cells from an experimental model of primary hypertension.

## 2. Development of Preglomerular Vascular Lesions

This section examines arterial stiffening and afferent arteriolar hyalinosis. In the kidney parenchyma, supplied by muscular arteries and arterioles, there is an accompanying progressive intimal thickening, as a part of the normal aging process. This thickening is greater on average among African American individuals than white individuals and it has a strong correlation with hypertension [1,10]. A progressive intimal thickening of small arterioles, that is arteriolosclerosis, occurs along development and progression of hypertension. Arcuate and interlobular arteries show intimal thickening. Smooth muscle cells, after changing into myofibroblasts, migrate from the media to the intimal layer and secrete collagen inducing intimal thickening. The intimal thickening is composed of several elements, including collagen and elastic fibers and myofibroblasts that have migrated in from the media, often with resulting thinning of the underlying media. Contrary to muscle cells in the media that have a circumferential arrangement, the media-migrated myofibroblasts are aligned along the long axis of the lumen. When contracting, longitudinally elongated myofibroblasts cause wall stiffness, with little or no effect on the lumen caliber [11].

### Afferent Arteriolar Hyalinosis

Hypertension also causes thinning of the media and, at the sites where smooth muscle cells are atrophic or missing, induces accumulation of hyaline material. This process, known as hyalinosis, is characterized by loss of smooth muscle cells and, therefore, should be distinguished from arteriolosclerosis. As a consequence of hyalinosis and cell loss, the wall of small vessels becomes more expansive, allowing the endothelial permeability and migration of plasma proteins to the media [12]. In hypertension, hyalinosis mainly entails the afferent arteriole leading to modest changes in the intraglomerular hemodynamics, whereas in diabetes hyalinosis affects the afferent and, to a larger extent, the efferent arteriole, thereby inducing an increase in the intraglomerular pressure [12]. 

Hyalinosis can be also found in ageing kidneys, indicating that hyalinosis is not a hallmark of hypertensive kidney disease [11,13]. However, in hypertensive patients, hyalinosis develops earlier and more markedly than in normotensive ageing, thus supporting the concept that hypertension causes an accelerated senescence [14]. Hyalinosis affects the glomerular arterioles, although in the latter plasma protein filtration is less pronounced than in the diabetic kidney. 

The arcuate and interlobular arteries and afferent arterioles are exposed to increasingly pulsatile flow at ever-widening pressures, changes that cause the progressive breakdown of elastic fibers in large arteries that are accentuated in hypertension.

In confirmation, isolated systolic hypertension with increased pulse pressure has been associated with microalbuminuria [13,15], strongly implying that the increased pressure reaches down to the glomerular level, and it is a factor in the progression of glomerulosclerosis.

## 3. Glomerular Damage 

In uncomplicated hypertensive nephrosclerosis there are basically three types of glomeruli: normal glomeruli, ischemic glomeruli and hypertrophic glomeruli [16,17]. Ischemic glomeruli show varying degrees of capillary collapse and retraction of the tuft, with filling of Bowman’s space with collagen as the glomerulus becomes sclerotic leading to glomeruli termed obsolescent [18]. In hypertrophic glomeruli with enlarged tufts and dilated capillaries, eventually lesions typical of focal segmental glomerulosclerosis (FSGS) are manifest with capsular adhesions, segmental scars and hyalinosis lesions. 

Normally, increases in blood pressure—episodic or sustained—result in proportionate autoregulatory vasoconstriction of the preglomerular resistance vessels, primarily the afferent arteriole, thus, renal blood flow (RBF), glomerular filtration rate (GFR) and glomerular capillary hydraulic pressure (PGC) are maintained relatively constant [19]. 

Most of the patients with primary essential hypertension have preserved autoregulation. The glomerular capillaries are protected from barotrauma and significant proteinuria is not seen. However, the preglomerular vasculature exposed to hypertension develops the slowly progressive vascular pathology of benign nephrosclerosis [19,20]. 

In hypertension, tightening of the afferent arteriole causes partial ischemia of the glomerular tuft that becomes smaller and gradually reduces the filtration [11]. The ischemic glomerulosclerosis and nephron loss that occur over time are usually not sufficient to result in ESRD [11]. Hyperfiltration and hypertrophy of the remaining nephrons allow the kidney to function longer, thereby explaining why early ESRD occurs in hypertensive patients if other risk factors are present, including obesity, smoking or diabetes mellitus. Although a subset of genetically susceptible individuals, largely African Americans, seem to exhibit a more accelerated course [21,22,23,24]. Histologic studies in such individuals have suggested a focal loss of autoregulation and barotraumas-mediated focal segmental glomerulosclerosis in addition to ischemic glomerulosclerosis [11,25]. The term benign decompensated nephrosclerosis has been used to describe such pathology. 

When hyalinosis is the major change affecting afferent arteriole in hypertension, glomeruli become hypertrophic [11,26,27] with enlargement of the glomerular tuft and occlusion of capillaries by hyaline material, leading to accumulation of periglomerular extracellular matrix (ECM) and FSGS [19,27]. The combination of dilated hyaline afferent arterioles serving glomeruli with enlarged tufts and increased size of the individual capillary loops points to possible loss of autoregulation in these glomeruli [11]. 

## 4. Podocyte Depletion

Mechanical forces take center stage among the factors accounting for podocyte loss. The major way of losing podocytes as viable cells consists of detachment from the glomerular basement membrane (GBM). The principal factor driving podocyte detachment is glomerular capillary hypertension which leads to increased circumferential and axial capillary wall stress as well as increased filtrate flow (i.e., hyperfiltration), conducting to enhanced fluid shear stress on the podocytes [28]. Hyperfiltration increases fluid shear stress, and glomerular hypertrophy compels podocytes to cover larger and more remote areas of GBM. Increased shear stress on podocytes in vivo is generally found under conditions of hyperfiltration (i.e., increased filtrate flow), which may be the consequence of increased glomerular plasma flow or glomerular hypertension. Hypertension will increase filtrate flow by increasing the driving force (increasing the filtration fraction) and expanding the GBM area [29].

Podocytes in vivo are exposed to shear stress at two sites: the foot processes at the level of the filtration slits and the primary processes and cell bodies within Bowman’s space. At these sites, the magnitude of the forces involved differs substantially. The filtration slits between foot processes are very narrow; at the level of the slit diaphragm, they are roughly 35–40 nm [30]. Kritz et al. hypothesized that the slit diaphragm membrane may subdivide the filtrate flow into many smaller flow streams, thereby diffusing the shear stresses acting on the lateral walls of the foot processes over a larger area. Shear forces acting on the individual protein components (e.g., nephrin) of the slit diaphragm may be transferred laterally to the podocyte cytoskeleton by the connections of the intracellular moieties of these molecules through linker proteins to the actin cytoskeleton. Either increased filtrate flow through the slit membrane or expansion of the slit membrane caused by capillary dilation (with possible increases in its pore size) would likely be tolerated only up to a certain point. If this tolerability limit is passed, the above mentioned signaling cascade to the actin cytoskeleton will tend to close the slit, replacing it by occludens-type junctions (i.e., starting foot process effacement), to limit the exposure of foot processes to untenable rheologic stresses [28]. The initial response of closing the slits seems to be the most critical for protection to acute mechanical forces. If it is successful, the completed stage of foot process effacement—with attachment of the soma to the GBM—may develop against detachment. Notwithstanding the protective responses to such increased rheologic stresses may fail, leading to retraction and broadening of foot processes or foot processes greatly deformed in shape and separated by relatively wide empty spaces of bare GBM and local detachment with formation of pseudocysts [31].

Glomerular hypertrophy leads to the defiance of the podocytes to cover increased and more remote areas of GBM. Such forms of hypertrophic overload include two aspects. First, the capillary areas that a given podocyte has to cover move apart from each other. Both seem unable to adequately cope by hypertrophy alone, with the overall tuft growth leading to a stretching of podocytes with cell body attenuation. This overload, in turn, is frequently accompanied by a narrowing of the outflow clefts from the subpodocyte spaces, resulting in pseudocyst formation when the outflow of the filtrate is impaired. Pseudocysts are seen to develop in response to unbalanced hypertrophy. Second, the adaptability of podocyte foot processes to hypertrophic growth may also have an upper limit, leading to increased shear stresses locally that may initiate the process of detachment and podocyte loss [32].

In advanced stages of disease, increased resistance to the outflow of filtrate from the sub-podocyte spaces will lead to expansile forces on podocytes, also favoring their detachment [28]. Intraglomerular pressure bulges outwards the denuded GBM, thereby favoring focal adhesions of the glomerular tuft to the outer leaflet of Bowman’s capsule and glomerulosclerosis [33]. Spreading of the lesion to a focal scar follows with misdirected filtration into the renal interstitium and ultimately spreading of primary glomerular injury into the renal tubulointerstitium [34].

## 5. Tubulointerstitial Fibrosis

The main feature of nephrosclerosis is interstitial inflammatory fibrosis. It has long been demonstrated that fibrosis entraps the peritubular capillaries and largely contributes to the decline in renal function [35]. In hypertensive patients, interstitial inflammatory fibrosis may develop a large spectrum of lesions in the epithelial tubular cells [36]. Lesions range from cell dilatation and flattening to atrophy and loss. Other patterns include flat cells surrounding widely open lumens filled with eosinophilic casts, known as ‘thyroid areas’, which are often associated with apolipoprotein L1 gene renal risk alleles [37]. 

Inflammatory cells may be spread, and may act as a trigger for tubulointerstitial fibrosis (TIF) [36]. Infiltrate of inflammatory cells results from both activation of resident inflammatory cells and recruitment of circulating inflammatory cells. There is an increase in interstitial fibroblasts due to increased proliferation and decreased apoptosis of resident interstitial cells at the tubulointerstitial space. Behind proliferation, these cells may undergo a phenotypic transformation and acquire fibrosis-promoting properties. This phenotypic change can be recognized by the acquisition of smooth muscle cell-type properties and de novo expression of α smooth muscle actin (α-SMA). Such α-SMA-expressing cells, myofibroblasts, restricted to periarteriolar regions in normal kidneys, densely populate the interstitium of chronically damaged kidneys. Myofibroblasts are the primary site of enhanced matrix synthesis. Compressed by collagen, peritubular capillaries become atrophic, and distance between tubular cells and capillaries increases, thereby worsening the damage and facilitating the development of renal failure [37]. Loss of the microvasculature implies a hypoxic milieu and suggests an important role for hypoxia. The ‘chronic hypoxia hypothesis’ was proposed By Fine and Norman [38]. The explanation supporting this hypothesis is that injury primarily determined at the glomerular level by hypertension causes changes in post-glomerular peritubular capillaries, which in turn induce endothelial damage and hypoxia. Microvasculature dysfunction, by inducing a hypoxic environment, triggers inflammation, EMT with myofibroblast differentiation and fibrosis [11,38,39]. Therefore hypertension-induced hypoxia translates the initial glomerular injury into interstitial damage [38]. Resident interstitial fibroblasts are not the only precursor cells, an important additional source of origin is tubular epithelial cells that have transdifferentiated into mesenchymal cells, being this process, epithelial–mesenchymal transition [40]. 

In vitro studies have largely focused on proximal tubular epithelial cells (PTECs) as the predominant epithelial cell type in the cortex and susceptible to hypoxic injury. 

In PTECs, hypoxia induces a complex transcriptional response with changes in expression of a large number of genes involved in cell survival and adaptation [41]. The hypoxia-inducible factor (HIF) family are considered the principal regulators of the adaptive response controlling expression of hundreds of genes. The master regulator of this adaptive response is hypoxia-inducible factor α (HIF-α), a heterodimeric transcription factor comprising a constitutively expressed beta-subunit and an oxygen-regulated alpha-subunit [42]. Under hypoxia, HIF-α protein dimerizes with HIF-β and binds to hypoxia-response elements in the regulatory regions of target genes coding for transforming growth factor β1 (TGFβ1), collagens and other ECM proteins leading to fibrosis [38]. In the hypoxic kidney, HIF-1α accumulates in tubules and in papillary interstitial cells whereas HIF-2α is induced in peritubular endothelial cells and fibroblasts. Hypoxia modifies proximal tubule epithelial matrix metabolism, promoting ECM accumulation with a switch to production of interstitial collagen and suppression of matrix degradation [43]. EMT is increasingly implicated in fibrosis; exposure of PTECs to hypoxia induces a myofibroblastic phenotype whereas more prolonged exposure leads to mitochondrial injury and apoptosis consistent with the loss of tubular cells in vivo [44,45]. Data from animal models provide a strong argument for hypoxia as a primary mediator of progressive scarring in the kidney, however a critical question is whether this also applies to hypertension in humans [46]. Although there are currently only limited clinical data, the relationship between rarefaction of peritubular capillaries and tubulointerstitial scarring in human biopsies strongly suggests a role of hypoxia in chronic kidney disease. Notwithstanding, a direct demonstration of the chronic hypoxia hypothesis in the hypertension nephropathy in humans is still lacking [38,47].

### Angiotensin II and Tubulointerstitial Fibrosis 

A well-known inducer of tubulointerstitial fibrosis is angiotensin II. This peptide, the most powerful vasoconstricting agent of the RAS, participates in local and systemic hemodynamic regulation, and it is also a true profibrotic cytokine. Angiotensin II plays a significant role in cell growth and proliferation, generation of reactive oxygen species, inflammation and extracellular matrix (ECM) synthesis and degradation. Many effects of angiotensin II are dependent on the angiotensin II type 1 receptor (AT_1_R) stimulation that leads to reactive oxygen species (ROS) production by NADPH oxidase. From the seven NADPH oxidase isoforms that have been identified, the NADPH oxidase 4 (Nox4) is abundantly expressed in kidney PTECs. ROS generated by NADPH oxidase can also act as signaling molecules that mediate angiotensin II–dependent signal transduction pathways (redox-sensitive signaling) [48,49]. Studies have suggested that caveolin 1 plays an important role as a platform for the compartmentalization of redox signaling events through NADPH oxidase–dependent production of ROS [50]. In spontaneously hypertensive rats (SHR), it has been demonstrated gene expression of the main components of phagocyte NADPH oxidase in renal cells, suggesting that the activation of NADPH oxidase within the kidney may precede the development of hypertension. Moreover, ROS function as signaling molecules and contribute to cell growth, migration, differentiation, and cytoskeletal remodeling [51] being the major pathway by which angiotensin II induces epithelial–mesenchymal transition and apoptosis leading to fibrosis. Previously, we showed that NADPH oxidase activity and Nox4 protein levels were increased in microdissected proximal tubule cell membranes from SHR, an effect which was prevented by losartan treatment. Interestingly, losartan markedly increased proximal tubular caveolin 1, suggesting that intrarenal angiotensin II is involved in the negative regulation of caveolin 1 during the development of hypertension and renal injury in SHR [52]. It has been shown that treatment with a specific NADPH oxidase inhibitor, apocynin, decreased NADPH oxidase activity and attenuated PTECs apoptosis and tubulointerstitial fibrosis in mice with proximal tubular cell–specific overexpression of angiotensinogen. These data are consistent with the concept that locally generated angiotensin II in proximal tubules induces renal injury through NADPH oxidase-dependent ROS production [53]. 

Zhou et al. have shown that angiotensin II activates nuclear transcription factor NF-κβ in proximal tubules, leading to long-lasting inflammatory and growth-promoting effects; these responses were blocked by inhibition of AT_1_ receptor–mediated endocytosis of extracellular angiotensin II with losartan [54]. The peptide angiotensin II is also involved in the inflammatory process through the synthesis of chemotactic factors, such as monocyte chemoattractant protein-1 (MCP-1) and by activation of resident cells by macrophage-related factors, and therefore contributes to the progression of fibrosis. Angiotensin II promotes the phenotypic change of fibroblasts to myofibroblasts (α-smooth muscle actin–positive cells). These activated fibroblasts may proliferate and invade the periglomerular and peritubular spaces, contributing to matrix deposition in the tubulointerstitial area [55].

A close connection between HDL (High density lipoprotein) and monocyte has been confirmed in studies which affirmed that the increased number of monocytes and HDL reduction may contribute to a major risk of plaque formation, progression of atherosclerosis and, consequently, an increased cardiovascular disease risk [56,57]. Recently, monocyte count to HDL-cholesterol ratio (MHR) has emerged as a potential marker of inflammation. Gembillo et al. demonstrated that MHR is linked with the use of multiple anti-hypertensive therapy and resistant hypertension in chronic kidney disease (CKD) patients [58].

In an experimental model of hypertension, SHR, one of the second-generation of AT_1_ blocker, telmisartan reduced albuminuria and proteinuria. However, the combination treatment with telmisartan and the statin pitavastatin displayed a more effective decrease in albuminuria and proteinuria, even to the normal level. The combination therapy decreased intrarenal angiotensin II and AT_1_ expression, in contrast to telmisartan or pitavastatin alone. In addition, adjunctive therapy with pitavastatin to telmisartan greatly attenuated the activity of the TGF-b/smad3 and NF-kB pathways and then inhibited renal fibrosis and inflammation leading to renoprotection [59].

Experimental and clinical evidence indicate that vitamin D deficiency and angiotensin II upregulation play a pivotal role in the progression of renal disease associated with hypertension [60]. The pleiotropic actions of vitamin D and its analogues are mediated by the vitamin D receptor (VDR), a ligand-dependent transcription factor that belongs to the steroid nuclear receptor gene family [61]. The interaction of plasma renin and vitamin D is tightly connected with the VDR status: in case of vitamin D deficiency there is a reduced transcription of VDR and an enhanced degradation of unliganded VDR, with a decrease in both unliganded and liganded VDR. This deficiency of liganded VDR, as previously mentioned, would improve the transcription of renin whereas the lack of unliganded VDR would enhance the transcription of angiotensinogen and AT_1_ receptors via modulation of p53 expression, as proved in an animal experiment conducted by Chandel et al. [62].

Thus, it appears that lack of VDR or VDR deficient state has at least two ways to activate the RAAS, and vitamin D could be a modulator of RAAS through VDR control [63]. Furthermore, VDR activators have not only suppressant effects on the renin–angiotensin system (RAS) but also anti-inflammatory and anti-fibrotic ones [64]. Therefore, calcitriol protects renovascular function in hypertension by downregulating AT_1_ receptors and reducing oxidative stress [65].

Rossi et al. in a transgenic model of severe hypertension and cardiovascular damage, the TG(mRen2)27 rat, created by the insertion of the mouse renin gene into the rat genome, found that either angiotensin-converting enzyme (ACE)-inhibition with ramipril [66], or the block of AT_1_R with AT_1_ receptor blocker irbesartan [67] prevented TIF, supporting the fibrogenic role of angiotensin II.

The pathogenic actions of the ACE/angiotensin II/AT_1_ axis can be countered by the angiotensin-converting enzyme 2 (ACE2)/angiotensin 1–7/Mas receptor (Mas) axis. As a homolog of ACE, the monocarboxypeptidase ACE2 can oppose ACE activity via conversion of angiotensin II to angiotensin 1–7, which binds to the Mas receptor to exert opposite effects on angiotensin II [68]. ACE2 is highly expressed in the normal kidney, mainly located in the proximal tubule, and plays an essential role in cardiovascular and kidney diseases [69].

Previously it has been reported that angiotensin II can induce a rapid activation of Smad2/3 which results in a subsequent expression of collagen I in tubular epithelial cells lacking the *TGF-β* gene. These profibrotic actions are blocked by the AT_1_ antagonist (losartan) and ERK1/2 inhibitor (PD98059) [70]. In addition, disruption of Smad3 prevents angiotensin II-induced kidney injury by preserving renal function, inhibiting renal fibrosis and inflammation, but has no effect on angiotensin II-induced high blood pressure in vivo [71]. These findings reveal that angiotensin II may act via the AT_1_-ERK1/2-Smad3 pathway to mediate renal fibrosis, which can be counter-regulated by angiotensin 1–7. Activation of Smad3 can then transcriptionally induce Smurf2, an E3 ligase, which binds and causes degradation of Smad7 protein [72,73].

It is also reported that Smurf2-mediated degradation of renal Smad7 protein plays a pathogenic role in the progression of tubulointerstitial fibrosis. Since Smad7 functions as an integrated inhibitor for both TGF-β/Smad–signaling and NF-κB–signaling pathways, once renal Smad7 is degraded, TGF-β/Smad and NF-κB signaling becomes activated, resulting in the development of renal fibrosis and inflammation [74]. Liu et al. have previously shown that Smad7 is renoprotective in hypertension, as deletion of Smad7 enhances whereas overexpression of renal Smad7 inhibits angiotensin II–induced hypertensive heart and kidney injury [73].

Mice lacking ACE2 developed more severe hypertension and hypertensive nephropathy, which was associated with a marked activation of the Smurf2-dependent Smad7 ubiquitin degradation pathway [75]. These findings show that angiotensin II may act via the AT_1_-ERK1/2-Smad3 pathway to mediate renal fibrosis, which can be counter-regulated by angiotensin 1–7. In addition, angiotensin II can also activate Smad3 by degrading Smad7, a downstream inhibitor of TGF-β/Smad signaling, via the Smurf2-dependent ubiquitin-proteasome degradation mechanism [76]. Thus, deletion of Smad7 promotes, but overexpression of Smad7 inhibits angiotensin II-induced AT_1_-ERK1/2-Smad3-mediated hypertensive nephropathy [77].

Consistent with this notion, Lui et al. demonstrated that a single deletion of ACE2 or Mas sustained AT_1_-NF-κB-driven renal inflammation and fibrosis, which became more severe in mice lacking both ACE2/Mas genes. Thus, enhanced AT_1_-ERK1/2-Smad3 signalling may be a mechanism by which loss of the ACE2/angiotensin 1–7/Mas axis exacerbated renal fibrosis in ACE2/Mas double KO mice. Furthermore, angiotensin II can also activate NF-κB by degrading Smad7, as Smad7 is capable of inducing IκBα, an inhibitor of NF-κBα [78].

Previously, in the kidneys of angiotensin II infused-mice a significant upregulation of TGFβRI and P-Smad3 has been demonstrated. MicroRNAs (miRNAs or miRs), small (about 22 nucleotides) noncoding RNAs regulate gene expression via inhibiting translation or guiding corresponding target mRNA cleavage. Ding et al. have shown that MiR-101a overexpression significantly suppressed the angiotensin II-induced increase of TGFβRI and p-Smad3. Hence, these data suggested that miR-101a may exhibit anti-fibrosis activities through inhibiting the TGF-β/Smad3 signaling pathway. Moreover, overexpression of miR-101a inhibited angiotensin II-stimulated up-regulation of pro-inflammatory cytokines and decreased the phosphorylation level of IκBα and p65, which meant that miR-101a also acted as an inhibitor of the NF-κB signaling pathway in renal inflammation [79].

## 6. Involvement of Heat Shock Protein 70 in the Antioxidative Action of Losartan in Proximal Tubule Cells 

A protective mechanism of cells against the ROS effects is the production of highly conserved proteins called heat shock proteins (Hsps). These are ubiquitously expressed molecular chaperones that help maintain and restore the normal function of cells against stress, a condition that dramatically increases their expression [80]. Hsp70 regulates a diverse set of signaling pathways through its interaction with proteins [81]. Hsp70s intracellular locations are the cytosol, the nucleus, and the mitochondria [82]. Members of the HSP70 family are called the “triage” chaperones because they play key roles in both protein folding and turnover [83]. Hsp70 induction is an early survival response elaborated by stressed cells to counter cellular damage and hasten recovery. As central components of the cellular protein surveillance network, these ATP-dependent chaperones are involved in a large variety of protein folding processes. They promiscuously interact with practically all proteins in their unfolded, misfolded, or aggregated states but do not interact with their folded counterparts. In addition, a limited set of native proteins is recognized by Hsp70 and regulated in their activity, stability, and/or oligomeric state [84]. Hsp70s interact with many different polypeptide con-formers: with unfolded polypeptides, for example during de novo folding at the ribosome or translocation through biological membranes; partially structured on-path folding intermediates; protein species in off-path misfolded states; and native protein assemblies and non-native polypeptide aggregates [85].

We focused on the study of the mechanism involved in the antioxidative effect of losartan, a pharmacological inhibitor of the AT_1_ receptor, on the genetic model of hypertension in rats spontaneously hypertensive rats, (SHR). We investigated the contributions of caveolin-1 and heat shock protein 70 (Hsp70) to the regulation of Nox4 expression in the proximal tubules of SHR. Due to the interaction of caveolin 1 and inducible Hsp70 (Hsp70i) after AT_1_ receptor blockade, we demonstrated the role of both proteins in AT_1_R regulation in cell membranes from SHR microdissected proximal tubules. Plasma membrane translocated Hsp70i and its colocalization with caveolin 1 could be involved in the mechanism responsible for losartan cytoprotective effect on proximal tubules by decreasing oxidative stress through the downregulation of NADPH oxidase subunit Nox4 [52]. Supporting our results, a functional interaction of Hsp70 with NAD(P)H: quinone oxidoreductase 1 (NQO1), a potent antioxidant that catalyzes two-electron reduction of various quinones, with NADH or NADPH as an electron donor, was previously found. This interaction of the chaperone was observed with wild-type NQO1*1 but not with the mutant NQO1*2, a point mutation in exon 6 of the human NQO1 gene. The association of inducible Hsp70 chaperone with NQO1*1 may play an important role in the stability and functionality of the NQO1 protein [86].

Later, we investigated how the inducible Hsp70 chaperone regulates Nox4 subunit trafficking and degradation in losartan-treated SHR PTECs. It is known that Hsp70 promotes the proteasomal degradation of proteins by recruiting the ubiquitin ligase CHIP, a key component of the chaperone-dependent quality control mechanism [87]. Client proteins are efficiently targeted by CHIP, particularly when they are partially or frankly misfolded, as is the case for most proteins binding to Hsp70 through exposed hydrophobic residues [88]. In this way, Nox4 is roughly divided in two large domains: an N-terminal cluster of hydrophobic membrane-spanning sequences, and a C-terminal protein domain [89]. 

We demonstrated that losartan induces increased Hsp70 expression and decreased oxidative stress through Nox4 protein levels reduction in PTECs from SHRs. The decreased Nox4 was not due to modifications at transcriptional level expression, it was as a result of the interaction of Hsp70 with its CHIP cochaperone complex that negatively regulates Nox4 protein levels through proteasomal degradation. Conversely, Hsp70i knockdown enhanced Nox4 protein levels, NADPH oxidase activity, and ROS generation in SHR PTECs, revealing that losartan was unable to abrogate angiotensin II effects on Nox4 expression and oxidative stress activity. Taken together, these losses of function data indicate that the inducible Hsp70 chaperone is directly involved in Nox4 regulation through the physical and functional interactions that come under the antioxidative effect of losartan [90].

## 7. Epithelial–Mesenchymal Transition 

The main function of the epithelial cell is to provide structure and prevent permeability; therefore, its most prominent characteristic is tight junctions and polarity. On the other hand, the prototype for mesenchymal cells is fibroblasts whose function is to deposit collagen as a repair mechanism often secondary to an insult. EMT is a phenomenon where inherent epithelial markers are lost and mesenchymal characteristics are gained. Simplistically speaking, an epithelial cell transitions into a mesenchymal cell, losing all the characteristics inherent to epithelial cells and gaining mesenchymal features in the process. The end result is a motile, collagen-producing cell from epithelial origin [91].

Thus, by providing a new source of fibroblasts, EMT becomes a possible contributor to fibrosis in end-stage organ disease and, as such, may become a new potential anti-fibrotic target. EMT has been described in many contexts such as in embryologic development, endocardial cushion formation, metastatic properties in breast cancer, and fibrotic response in interstitial pulmonary fibrosis. Its role is probably more extensive than what is previously thought. In recent years EMT has been found to play a crucial role in fibrosis, in particular in the kidney [92]. The main inducers of EMT are TGFβ1 and angiotensin II, the same factors that classically induce fibrosis, suggesting that EMT is a common mechanism underlying TIF [93]. 

Recently, Seccia TM et al. found that endothelin-1 can induce EMT in the kidney from an experimental model of Transgenic (mRen2)27 rat [TGRen2], developing fulminant angiotensin II–dependent hypertension. In the kidney sections of (mRen2)27 rats, the authors found a decrease in the epithelial marker E-cadherin along with an increase in the mesenchymal markers α-SMA and S100A4, a hypoxia inducible gene in the sites of TIF, suggesting EMT as the mechanism underlying fibrosis. Renal TIF was prevented in (mRen2)27 rats not only by the AT_1_ receptor blocker-irbesartan, but also by bosentan, an antagonist of endothelin-1 system [67,94].

## 8. Angiotensin II and Epithelial–Mesenchymal Transition 

Angiotensin II has been directly implicated in tubulointerstitial injury and fibrosis, [55,95,96]. Several studies showed that angiotensin II induces the expression of mesenchymal markers in experimental models of renal fibrosis. Johnson et al. showed that angiotensin II infusion inducing mild-to-moderate hypertension in the rats was associated with interstitial cell proliferation [97]. The phenotype changes in which numerous interstitial cells expressed α-SMA and deposition of type IV collagen lead to fibrosis formation. It appears likely that the renal interstitial “fibroblast” becomes activated in angiotensin II-mediated injury, assuming characteristics of “myofibroblasts” in that these cells transiently proliferate, express α-SMA, and secrete type IV collagen.

In cell culture models, angiotensin II has been shown to induce tubular EMT. Burns et al. have demonstrated that angiotensin II induces EMT in a rat proximal tubular cell line (NRK52-E) as defined by changes in cellular morphology from the typical cobblestone pattern of the epithelial cell in culture to elongated, spindle-shaped mesenchymal cells. This transition was associated with a reduction in E-cadherin protein and de novo synthesis of α-SMA protein expression [98].

We investigated the role of Hsp70 through losartan effect on angiotensin II induced epithelial–mesenchymal transition in primary culture of PTECs from a genetic experimental model of hypertension (SHR). We demonstrate that Hsp70 within the losartan effect inhibits the EMT induced by angiotensin II in PTECs from SHR. Losartan increased E-cadherin levels and decreased vinculin, α-SMA, vimentin, pERK, p38 and Smad2–3 activation compared to angiotensin II treated and without treatment SHR PTECs. Moreover, losartan inhibited matrix metalloproteinase-2 and 9 secretion, reduced migration and cellular displacement, stabilizing intercellular junctions. Notably, losartan treatment in shHsp72 knockdown SHR PTCs showed results similar to SHR PTECs that lead to the EMT process. Losartan treatment in SHR PTECs induced decrease in Rac1 and RhoA activation. Although, in shHsp72 Knockdown SHR PTECs no significant changes were observed [99].

**Figure 1:**
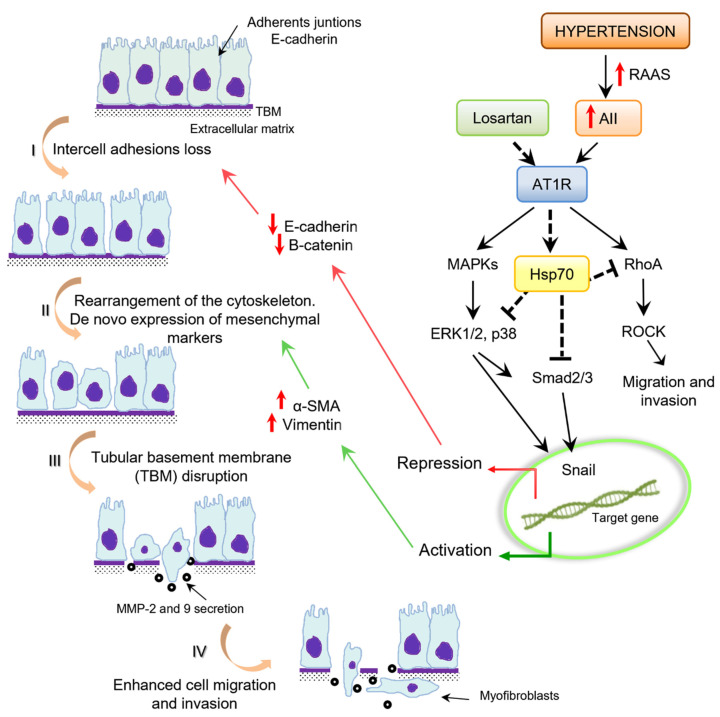
Proposed mechanism by which heat shock proteins 70 KDa (Hsp70) mediates the epithelial–mesenchymal transition (EMT)-negative regulation after angiotensin II type 1 receptor (AT_1_R) blockade by losartan in proximal tubule epithelial cells (PTECs) from spontaneously hypertensive rats (SHR). Left: key events during tubular epithelial cells to myofibroblast transition. Right: The Hps70 inhibits the signaling pathways that lead to EMT after losartan AT_1_ receptor blockage.

According to these results, Rodrigues-Díez et al. [93] demonstrated that RhoA inhibition through transient transfection of a dominant negative RhoA or the use of ROCK inhibitors regulates angiotensin II-mediated EMT in angiotensin II treated human proximal tubule cells (HK2). In our study, we demonstrated that losartan avoids the epithelial mesenchymal transition induced by angiotensin II, and we characterize the Hsp70 chaperone as an important mediator of the losartan effect which entails migration reduction and cytoskeletal stabilization in proximal tubule cells (Figure 1). We focus on this chaperone as a potential target that ameliorates renal damage.

Our data indicate that Hsp70 is involved in the cellular response after AT_1_ receptor blockage, inducing decreased profibrotic factors that lead to mesenchymal–epithelial transition. 

## 9. Conclusions

**Figure 2:**
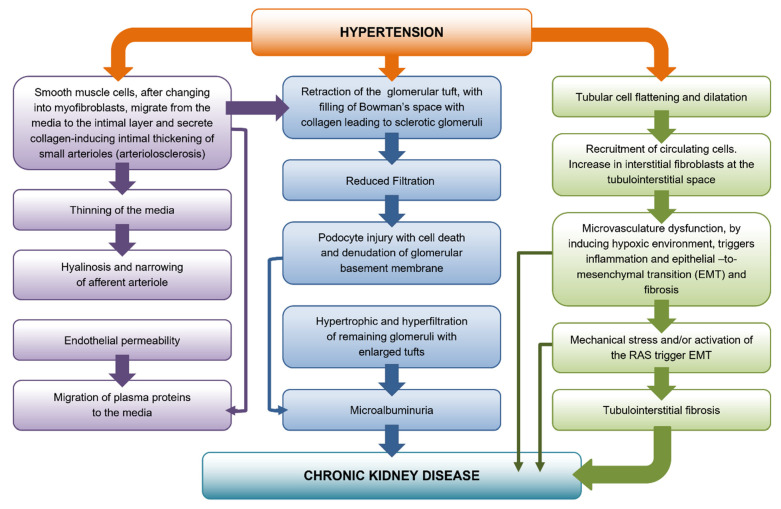
Histopathology and molecular mechanisms underlying nephropathy in primary (essential) hypertension. Modification in the vascular, glomerular and tubular compartments lead to the development of chronic kidney disease.

Hypertensive nephrosclerosis involves hyalinization and sclerosis of interlobular and afferent arterioles, together with glomerular and tubulointerstitial compartments fibrosis (Figure 2). Age and hypertension-related arterial stiffening mean that blood is delivered to afferent arterioles at increased pressure. Glomerular ischemia leads to hypoxia in the postglomerular vasculature at an early stage with resulting predominantly monocytic/macrophagic inflammation and EMT. For years, hypertensive kidney disease has been considered as a disorder limited to afferent arterioles and glomeruli in which mechanical stress induced by hypertension, RAS stimulation and activation of resident fibroblasts were considered to be the pathogenic mechanisms underlying the kidney damage. Up to date, podocyte damage and loss, epithelial–mesenchymal transition and tubulointerstitial fibrosis have emerged as main processes for the development of cytoprotective therapeutic strategies. Our data suggest a cytoprotective role of the Hsp70 chaperone on the angiotensin II–induced epithelial mesenchymal transition after AT_1_ receptor blockage. Future investigations are needed to study the role of Hsp70 in combining new therapeutic strategies to block the epithelial–mesenchymal transition process preventing renal interstitial fibrosis.

## Data Availability

Not applicable.

## Abbreviations

ESRD	end-stage renal disease
BP	blood pressure
CKD	chronic kidney disease
RAS	renin–angiotensin system
EMT	epithelial–mesenchymal transition
Hsp 70	heat shock proteins 70 KDa
ECM	extracellular matrix
GBM	glomerular basement membrane
FSGS	focal segmental glomerulosclerosis
RBF	renal blood flow
GFR	glomerular filtration rate
PGC	glomerular capillary hydraulic pressure
TIF	tubulointerstitial fibrosis
PTECs	proximal tubular epithelial cells
ROS	reactive oxygen species
AT_1_R	angiotensin II type 1 receptor
SHR	spontaneously hypertensive rats
VDR	vitamin D receptor
TGFβRI	serine/threonine kinase receptor of TGF-β/Smad signaling
